# Comparison of Brain Activation Between Different Modes of Motor Acquisition: A Functional Near‐Infrared Study

**DOI:** 10.1002/brb3.70238

**Published:** 2025-01-08

**Authors:** Meng‐Hsuan Tsou, Pei‐Yun Chen, Yi‐Ting Hung, Yong‐Wei Lim, Shiuan‐Ling Huang, Yan‐Ci Liu

**Affiliations:** ^1^ School and Graduate Institute of Physical Therapy, College of Medicine National Taiwan University Taipei Taiwan; ^2^ Taipei First Girls High School Taipei Taiwan; ^3^ Physical Therapy Center National Taiwan University Hospital Taipei Taiwan

**Keywords:** functional near‐infrared spectroscopy, motor execution, motor imagery, action observation, mirror visual feedback

## Abstract

**Background:**

Different modes of motor acquisition, including motor execution (ME), motor imagery (MI), action observation (AO), and mirror visual feedback (MVF), are often used when learning new motor behavior and in clinical rehabilitation.

**Purpose:**

The aim of this study was to investigate differences in brain activation during different motor acquisition modes among healthy young adults.

**Methods:**

This cross‐sectional study recruited 29 healthy young adults. Participants performed a functional reaching and grasping task under ME, MI, AO, and MVF mode with their right arms at a frequency of 0.5 Hz for 1 min per task. Each task was performed three times in a random order. Brain activation in the supplementary motor area (SMA), premotor cortices (PMC), and primary motor cortices (M1) during tasks was measured using functional near‐infrared spectroscopy through 16 source‐detector channels.

**Results:**

ME showed significant activation in bilateral PMC, M1, and right SMA, with higher activation in the contralateral M1. MI induced greater activity in the PMC and SMA, particularly in the ipsilateral regions. MVF resulted in significant activation in bilateral PMC, SMA, and M1. AO showed an increasing trend in brain activation, but no significant differences in any channels. Compared to AO, ME and MVF induced significantly greater brain activity in M1.

**Conclusion:**

Activation levels under MI and MVF were comparable to that of ME. MI and MVF induced greater activity in the PMC and SMA, and MVF showed significant activity in all brain areas, especially in the bilateral M1. These findings support the application of different motor acquisition strategies according to individual needs. When ME cannot be executed, such as for individuals with hemiparesis or severe impairments of both upper extremities, MI and MVF may be applied, respectively, to drive neuroplastic changes.

## Graphical abstract

This study compared brain activation in healthy adults during four motor acquisition modes: Motor execution (ME), motor imagery (MI), action observation (AO), and mirror visual feedback (MVF), using functional near‐infrared spectroscopy (fNIRS). Significant activation in the primary motor cortex (M1), supplementary motor area (SMA), and premotor cortex (PMC) during ME, MI, and MVF, with the greatest activity in ME and MVF. MI and MVF offer viable alternatives to ME in rehabilitation settings where ME is impaired.

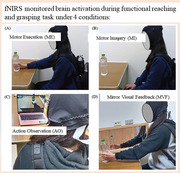

**Abbreviations**
AO: action observation
CBSI: correlation‐based signal improvement
fMRI: functional magnetic resonance imaging
fNIRS: functional near‐infrared spectroscopy
Hbdiff: hemoglobin differential
HbO: oxygenated hemoglobin
HbR: deoxygenated hemoglobin
M1: primary motor cortex
ME: motor execution
MI: motor imagery
MMSE: Mini–Mental State Examination
MNS: mirror neuron system
MVF: mirror visual feedback
PET: positron emission tomography
PMC: premotor cortex
SM1: sensorimotor cortex
SMA: supplementary motor area

## 1 Background

Human action and behavior are essential for completing tasks and learning new skills. Motor behavior encompasses observing, mimicking, and executing movements, while motor learning refers to the process of acquiring new behaviors through practice and observation. Specifically, motor acquisition involves the attainment of new motor skills, and motor execution (ME) is the physical performance of a motor task, which is a fundamental method for learning new motor behaviors.

To enhance motor learning beyond ME, alternative strategies such as motor imagery (MI), action observation (AO), and mirror visual feedback (MVF) are employed (Mulder 2007). MI involves mentally rehearsing movements without physical execution, commonly used by musicians to master difficult passages (Lotze 2013). Mirror therapy (MVF) utilizes visual stimulation by placing the affected limb behind a mirror, creating the illusion of normal movement through reflection (Thieme et al. 2018; Ramachandran and Rogers‐Ramachandran 1996). AO involves observing actions presented via video or performed by others (Mulder 2007; Small, Buccino, and Solodkin 2013; B. Zhang et al. 2019). This approach can aid in learning of complex movement sequences without physical training and has been shown to improve upper limb motor functions in individuals with stroke (B. Zhang et al. 2019; Cross et al. 2009). Initially, MVF was used to treat phantom limb pain in amputees and has proven effective in reducing pain and synesthesia (Ramachandran and Rogers‐Ramachandran 1996). MI, AO, and MVF are now widely applied in rehabilitation settings, particularly for conditions such as stroke, Parkinson's disease, and spinal cord injury, where ME is impaired due to neurological damage (Abbruzzese et al. 2016; Mateo et al. 2015; Cacchio et al. 2009).

Most previous studies investigated the brain activity during the different modes of motor acquisition individually, often using functional magnetic resonance imaging (fMRI) and/or positron emission tomography (PET) (Szameitat, Shen, and Sterr 2007; Jastorff et al. 2010; Jeannerod 2001). These early studies focused on the neural correlates associated with each mode, especially emphasizing the role of the primary motor cortices (M1) during ME. Studies investigating MI and AO individually using fMRI indicated involvement of the PMC and SMA, cingulate cortex, parietal cortical areas, basal ganglia, and cerebellum (Hanakawa et al. 2003; Tong et al. 2017). Later research compared brain activity between MI or AO and ME, finding that motor‐related regions, including the PMC and inferior parietal lobes, were activated during MI and AO. These studies also revealed that MI and AO share similar neural substrates with ME, though MI and AO tend to induce greater bilateral activation, while ME typically leads to stronger unilateral brain activity (Jeannerod 2001; Mizuguchi and Kanosue 2017; Crammond 1997; Condy et al. 2021; Miguel et al. 2021). A recent meta‐analysis comparing ME, MI, and AO found that all three modes activate the premotor‐parietal and somatosensory networks, with MI showing greater recruitment of the regions that are also involved in ME (Hardwick et al. 2018). Studies exploring brain activation under MVF have been more limited but suggest that MVF promotes activation of M1 contralateral to the limb reflected in the mirror (Altschuler et al. 1999; McCabe et al. 2003; Ramachandran and Altschuler 2009). While these imaging studies provide detailed information about deep brain structures, they impose restrictions on movement due to their need for participants to remain stationary, which limits the ecological validity of motor tasks.

More recently, portable and real‐time neuroimaging techniques such as electroencephalogram (EEG) and functional near‐infrared spectroscopy (fNIRS) have gained popularity for observing neural correlates during functional motor tasks without the need for fixed positions (Huo et al. 2019). EEG studies have shown that alpha and beta bands desynchronize over the sensorimotor cortex (SM1) contralateral to the imagined movement (Pfurtscheller and Neuper 1997; Pfurtscheller et al. 2005), and alpha band desynchronization was found over central‐parietal regions during AO (Babiloni et al. 2002; Calmels et al. 2006; Calmels, Jarry, and Stam 2009). A systematic review also demonstrated that AO or MVF enhanced mu suppression over the SM1 (J. Zhang et al. 2018). Considering the relatively higher spatial resolution and lower sensitivity to motion artifacts compared to EEG, fNIRS may serve as an alternative method for monitoring the brain activities during functional movements. Like fMRI, fNIRS measures changes in hemoglobin concentration, providing a comparable, yet more flexible, method for observing cortical activity during motor tasks (Su et al. 2023).

Recent studies have demonstrated the potential of fNIRS for capturing brain activity during motor tasks. For example, greater increases in oxyhemoglobin (HbO) levels have been observed during ME compared to MI (Batula et al. 2017). Qiu et al. used fNIRS to study brain activity during MVF with a robotic hand rehabilitation system, finding increased activation in the right PMC during MVF compared to a visual task without a mirror. In addition, combining MVF with the robotic rehabilitation system task led to higher activation in the SM1 compared to MVF alone (Qiu et al. 2022).

Despite the limitations in spatial resolution, fNIRS's balance of temporal resolution, portability, and its ability to measure cortical activity during naturalistic movements offer a valuable tool for exploring motor acquisition processes in functional settings. Moreover, most previous studies examined motor acquisition modes individually or compared them only to ME, without systematically comparing cortical activation patterns across multiple modes during upper limb functional movements. Few studies have explored brain activation during MVF in depth.

This study aims to investigate differences in brain activation during four motor acquisition modes (ME, MI, AO, and MVF) during a functional reaching and grasping task in healthy young adults. Understanding these cortical activation patterns may inform future clinical rehabilitation strategies for individuals with neurological impairments, with fNIRS providing a practical method for real‐time brain monitoring in dynamic, real‐world conditions.

## 2 Methods

### 2.1 Study Design

This cross‐sectional study explored differences in cortical activation patterns during an upper limb forward reaching and grasping task under four modes: ME, MI, AO, and MVF. The study protocol was approved by the National Taiwan University Hospital Institutional Review Board (NTUH IRB), and written informed consent was obtained from all participants.

### 2.2 Participants

Inclusion criteria for participants were as follows: (1) aged between 20 and 30 years; (2) right‐hand dominance, determined by the hand used to sign their name; (3) no history of neurological, cardiovascular, psychological, or other physical conditions that could interfere with study participation; and (4) intact global cognition, as indicated by a Mini–Mental State Examination (MMSE) score of 24 or above. Baseline characteristics, including age, gender, height, weight, dominant hand, and arm length, were collected at the start of the experiment.

### 2.3 Forward Reaching and Grasping Task

Participants were seated upright while performing a forward‐reaching and grasping task for 60 s under four different modes: ME, MI, AO, and MVF. Each mode was repeated three times in random order, for a total of 12 blocks, with a 40‐s rest between each block. The entire experiment lasted approximately 21 min.

Ten seconds before the start of each block, the research assistant provided verbal instruction for the upcoming mode (ME, MI, AO, or MVF). A metronome set at 0.5 Hz signaled the start of each block, and participants began the forward reaching and grasping task, following the metronome cue throughout the block. The distance of the object (a water bottle) placement was individualized based on each participant's upper limb length, calculated as 80% of their arm length. fNIRS data were continuously collected throughout the experiment.

#### 2.3.1 Motor Execution

**FIGURE 1:**
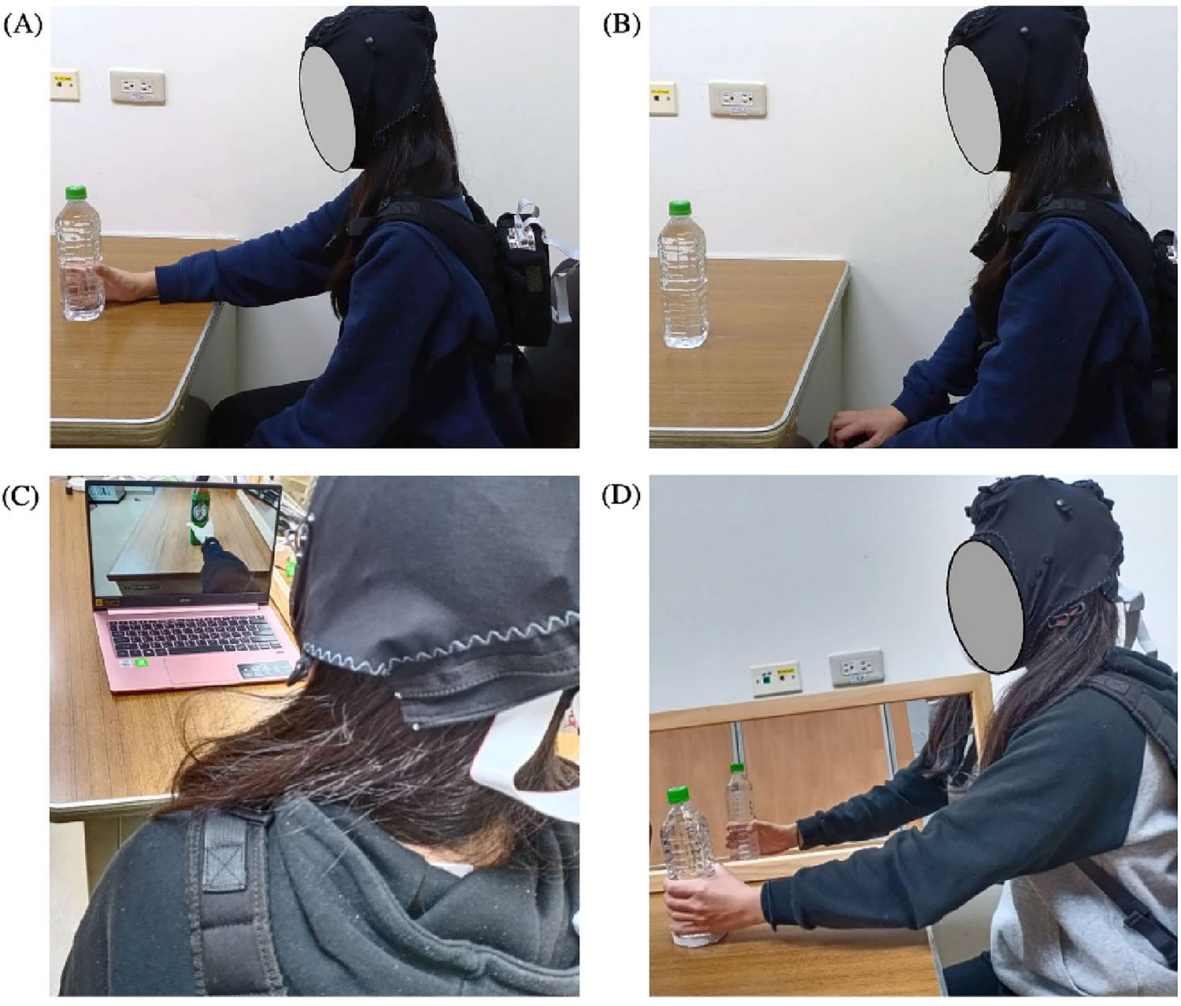
Foreword reaching task under the four different modes: (A) Motor execution (ME), (B) motor imagery (MI), (C) action observation (AO), and (D) mirror visual feedback (MVF).

Participants were instructed to use their dominant hand (right hand) to reach forward and grasp the water bottle placed on the desk in front of them. They were then to release their grip and bring their arm back down to their side, following the sound of a metronome (Figure 1A).

#### 2.3.2 Motor Imagery

Participants were instructed to gaze at the water bottle placed on the desk in front of them and perform a MI task. Specifically, they were to imagine reaching forward with their right arm to grasp the water bottle, then releasing it and bringing their arm back to their side (Figure 1B). This task involved visual MI, as participants were asked to visualize the movement from an external perspective while observing the water bottle.

#### 2.3.3 Action Observation

Participants were instructed to observe a video in which an individual uses their right arm to reach forward, grasp a water bottle, then release it and return the arm to the starting position. The video was filmed from the participant's first‐person perspective. Throughout this task, participants were instructed to remain seated quietly and upright, as shown in Figure 1C.

#### 2.3.4 Mirror Visual Feedback

A mirror was positioned in front of the participant, creating a separation between the right and left arms (Figure 1D), with the mirror reflecting the participant's non‐dominant arm (left arm). The research assistant ensured that the participant could see their left arm clearly in the mirror. Participants were instructed to keep their right arm relaxed and immobile on the opposite side of the mirror. Following the metronome cue, participants reached forward with their left arm to grasp a water bottle, then released it and returned their left arm to their side. Throughout this task, participants were asked to observe the movement of their arm in the mirror and to imagine that it was their right arm performing the movement.

### 2.4 fNIRS System and Placement

**FIGURE 2:**
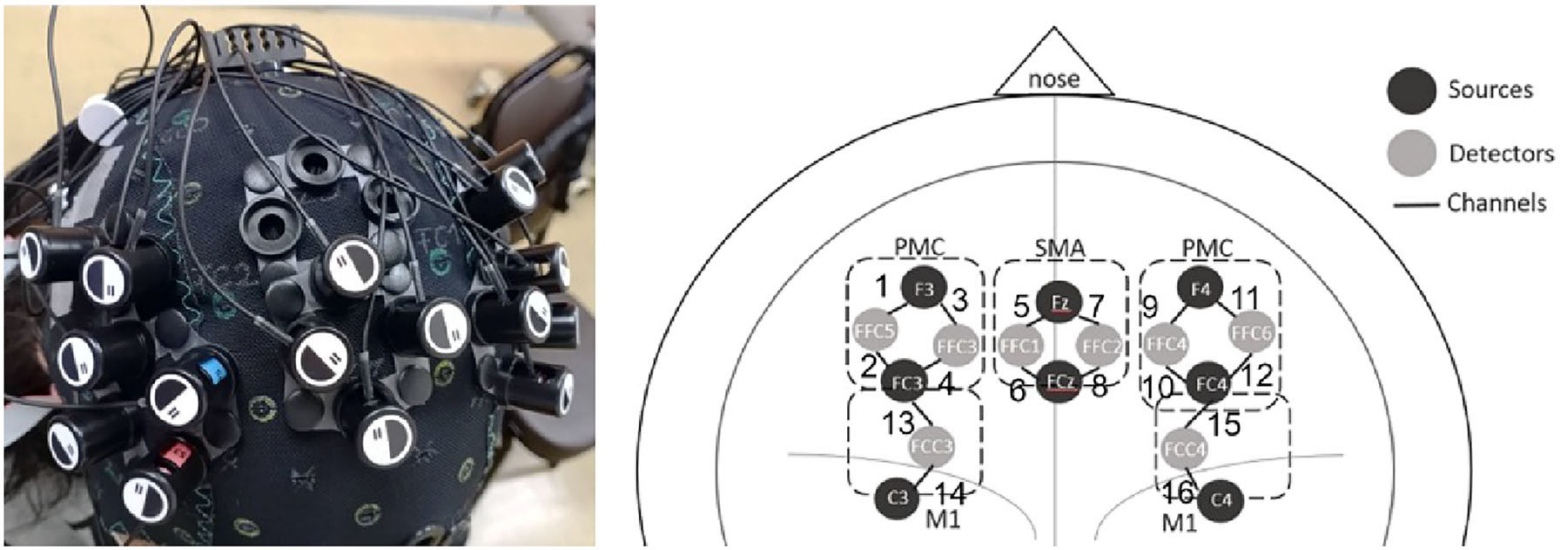
fNIRS optodes placement in the supplementary motor area (SMA), bilateral premotor cortices (PMC), and bilateral primary motor cortices (M1). The number on the source‐detector pathways represent the channel numbers.

A multichannel wearable fNIRS imaging system (NIRSport 2, NIRx Medical Technologies LLC, Glen Head, NY, USA) with eight LED light sources and eight detectors (dual wavelengths of 760 and 850 nm) was used to investigate cortical activation in this study. The fNIRS instrument exports and receives near‐infrared signals via LED optodes that are attached and secured to the participants’ heads by the fNIRS headcap. The headcap, which is designed to be compatible with the international 10–5 system, defines the standard surface positions of the human head with approximately 3.0 cm between any two adjacent optode positions (Oostenveld and Praamstra 2001). The eight sources and eight detectors, distributed across the bilateral primary motor cortices (M1), premotor cortices (PMC), and supplementary motor areas (SMA), formed 16 distinct channels. This configuration allows each detector to capture signals from multiple sources, and likewise, each source can be detected by multiple detectors, creating various source‐detector pathways. These pathways collectively defined the 16 channels used to monitor hemodynamic changes during the forward reaching and grasp task under each mode (ME, MI, AO, and MVF). Optode placement is illustrated in Figure 2.

The fNIRS base unit was placed in a backpack worn by the participants. The backpack straps were adjusted for each participant to ensure maximum comfort. The entire fNIRS system weighed less than 1 kilogram, and as participants were in the seated position, the base unit had minimal influence on motor performance.

### 2.5 fNIRS Data Analysis

The relative coefficient of variation ($\mathrm{CV}=\frac{\sigma }{\mu }\;\times 100\%$) was calculated for the raw signals at 760 and 850 nm to estimate the signal‐to‐noise quality of a data channel (Liu et al. 2018; Liu et al. 2022). Data rejection based on two types of CV, CV_chan_, and CV_trial_, was used to reduce physical artifacts, such as motion‐induced instability and blood pressure‐induced hemodynamics (Lu et al. 2015). CV_chan_ was calculated for each channel over the entire duration of the experiment (21 min), and CV_trial_ was obtained for each channel for the duration of each individual trial (1 min) for all four modes. Channels with CV_chan_ > 15% or CV_trial_ > 10% were rejected. The remaining fNIRS signals were bandpass‐filtered (cutoff frequency at 0.01–0.2 Hz) to eliminate the effects of heartbeat, respiration, and low‐frequency signal drifts for each wavelength (Piper et al. 2014). The preprocessed signals were then converted to concentration changes in oxygenated hemoglobin (HbO) and deoxygenated hemoglobin (HbR) by the modified Beer–Lambert law (Kocsis, Herman, and Eke 2006; Cope and Delpy 1988; Boas et al. 2001). Correlation‐based signal improvement (CBSI) was then employed to improve the signal quality based on the findings that brain activation involves HbO increases and HbR decreases in the activated cortical regions (Batula et al. 2017). The relative changes in HbO and HbR concentrations were calculated based on a 10‐s baseline and collected during the task of functional arm reaching.

The preprocessing of fNIRS signals, including the motion artifact correction, bandpass filtering, and conversion of HbO and HbR, was processed using the HOMER2 package (Altschuler et al. 1999). Neuronal activity typically results in a rapid increase in HbO and a less pronounced decrease in HbR based on the mechanisms of neurovascular coupling (Leff et al. 2011; Hoshi, Mizukami, and Tamura 1994). Therefore, we also calculated the hemoglobin differential (Hbdiff = HbO − HbR). In this study, we used HbO level as the primary indicator of fluctuations in regional cerebral blood volume, as it reflects changes in both regional cerebral blood volume and oxygenation, which are closely associated with neural activation. Research indicates that increases in HbO levels are sensitively correlated with increased brain activity across various tasks (Hoshi, Kobayashi, and Tamura 2001; Iso et al. 2021). Consequently, our analysis focused on HbO to provide a clear understanding of brain activity.

**FIGURE 3:**
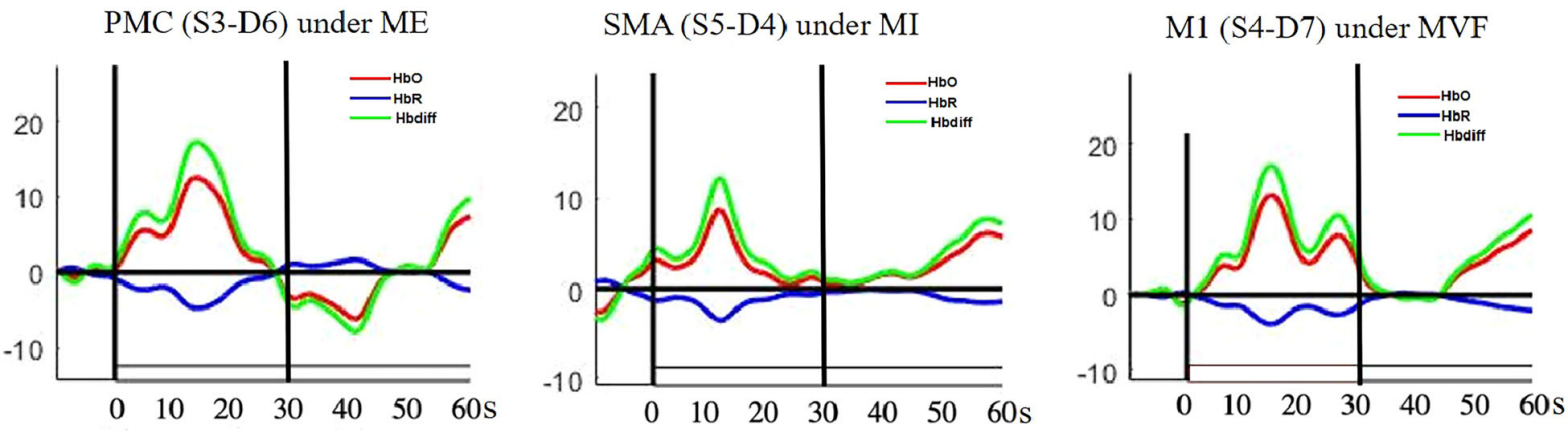
The averaged dynamics of HbO (red curves), HbR concentrations (blue curves), and Hbdiff level (green curves) during the “early phase” (0–30 s after task onset) and “late phase” (40–60 s after task onset). The horizontal solid lines depict the concentration level of zero, and the vertical solid lines label the time of zero for the task onset.

During the 60‐s functional reaching block, cerebral hemodynamics showed activation approximately 30 s after task onset, followed by relatively reduced or stabilized activation. Therefore, the first 30 s after task onset was defined as the “early phase,” reflecting the immediate hemodynamic response to the functional reaching task across all modes. The period from 30 to 60 s was defined as the “late phase” to assess sustained activation (Figure 3). A customized script developed in Matlab2017b (Matlab, The MathWorks Inc, Natick, MA) was used to process the fNIRS data for further statistical analysis.

### 2.6 Statistical Analysis

Descriptive statistics were generated for all variables and presented in means and standard deviations. A one‐sided *t*‐test with false discovery rate correction (FDR, *q* = 0.05) of multiple comparisons for the 16 channels was used to analyze differences in cortical activation under each mode and during each phase. One‐way repeated‐measures multivariate analyses of variance were used to assess differences in cortical activation patterns between each mode. A post hoc test with the Bonferroni correction was used for pairwise comparisons. Level of significance was set at *p* < 0.05.

## 3 Results

### 3.1 Participants

Twenty‐nine healthy young adults (13 males and 16 females) participated in this study. The mean age was 25.4 ± 3.0 years, the mean MMSE score was 29.1 ± 1.4, and all participants were right‐hand dominant.

### 3.2 Brain Activation

#### 3.2.1 Brain Activation During ME, MI, AO, and MVF

Brain activation results during early phase in different channels under four motor acquisition tasks was shown in Figure 5 and Table 1. During ME (Figure 4A), the right SMA (Ch7 and Ch8), bilateral PMC (Ch1, Ch10, Ch11, and Ch12), and bilateral M1 (Ch14, Ch15, and Ch16) showed significant activation in the early phase after task onset. In particular, the left M1 (Ch14) exhibited a trend of greater activation compared to the right M1 in the first 30 s of the functional reaching task.

FIGURE 4Brain activation during the four different modes: (A) motor execution (ME), (B) motor imagery (MI), (C) action observation (AO), (D) mirror visual feedback (MVF). The *t* values of significant activations with FDR correction for the multiple comparison in early or late phase are color‐coded under the axis for each channel. The horizontal solid lines depict the concentration level of zero, and the vertical solid lines label the time of zero for the task onset.

**Figure:**
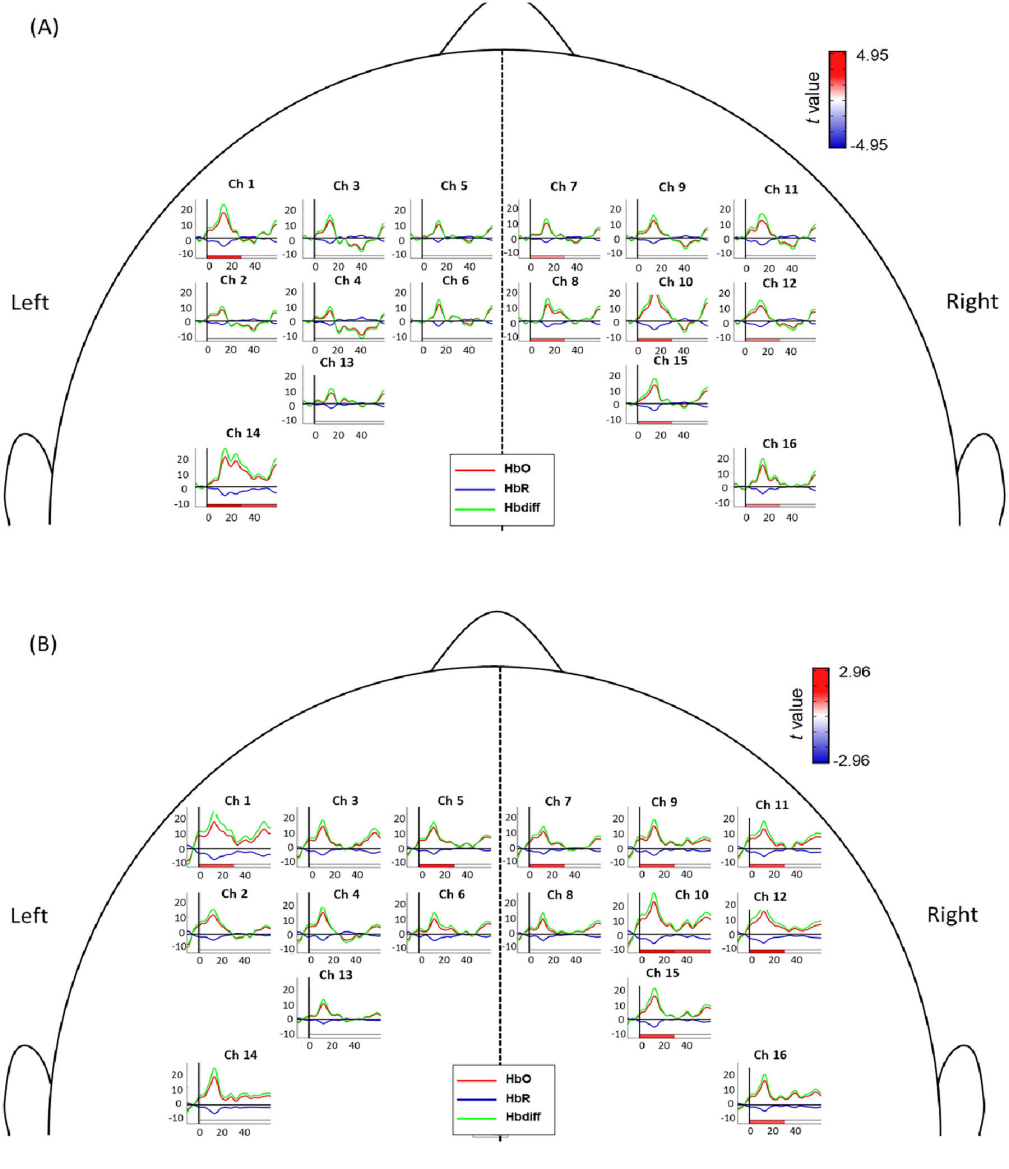

**Figure:**
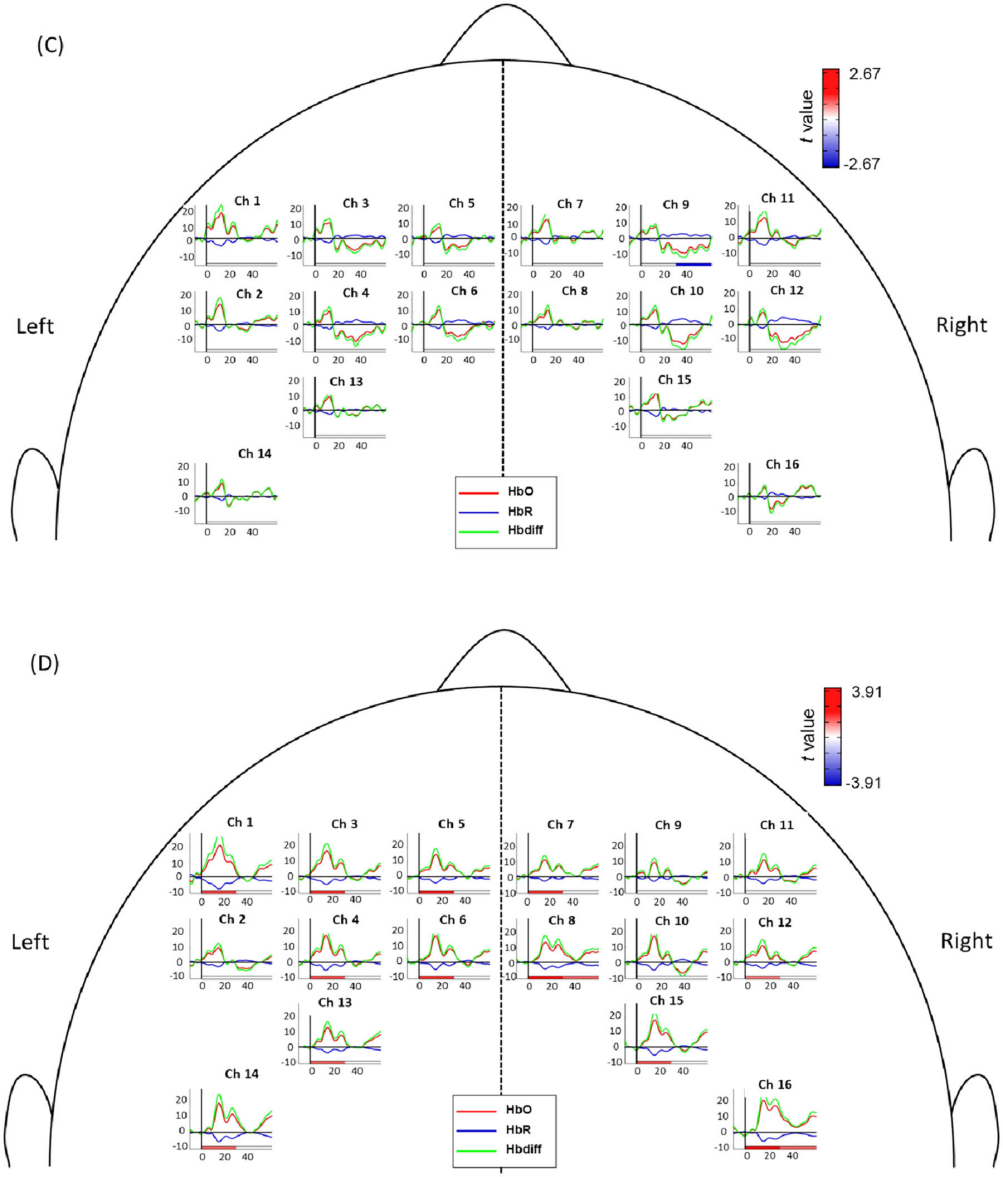

During MI (Figure 4B), significant activation was found in the bilateral SMA (Ch5 and Ch7), bilateral PMC (Ch1, Ch9, Ch10, Ch11, Ch12), and the right M1 (Ch15 and Ch16) in the early phase. The right PMC (Ch9, Ch11, Ch10, Ch12) showed greater activity compared to the left PMC.

During AO (Figure 4C), although all channels showed an upward trend after task onset, HbO and Hbdiff levels exhibited an immediate decrease in activity after approximately 15–20 s. Brain activation levels were not significantly different in any of the brain areas during AO.

During MVF (Figure 4D), significant activation was observed in almost all channels of the left PMC (Ch1, Ch3, Ch4), left M1 (Ch13, Ch14), and bilateral SMA (Ch5, Ch6, Ch7, Ch8) in the early phase after task onset. Minimal areas in the right PMC (Ch12) and right M1 (Ch15, Ch16) also showed significant brain activity. Statistical analyses confirmed significant activation in the bilateral SMA (Ch5, Ch6, Ch7, Ch8), bilateral PMC (Ch1, Ch3, Ch4, Ch12), and bilateral M1 (Ch13, Ch14, Ch15, Ch16).

#### 3.2.2 Comparison of Brain Activity Between Different Modes

We compared brain activation between each of the four modes of motor acquisition (Figure 5 and Table 1). Comparisons between ME and MI, ME and AO, and ME and MVF can be seen in Figure 5A–C. Activation patterns during MI and MVF closely resembled the activation pattern during ME, and statistical analyses revealed no significant differences between ME and MI or between ME and MVF (Figure 5A,C).

FIGURE 5Comparison of brain activation patterns between different modes: (A) ME and MI, (B) ME and AO, (C) ME and MVF, (D) MI and AO, (E) MI and MVF, (F) AO and MVF. The *p* values of significant activations with Bonferroni correction during early (0–30 s after task onset) or late (30–60 s after task onset) phase are color‐coded under the axis for each channel. The *x*‐axis depicts the time since the task onset and the *y*‐axis depicts the hemoglobin level. The hemoglobin level is shown, which was calculated by average of all participants.

**Figure:**
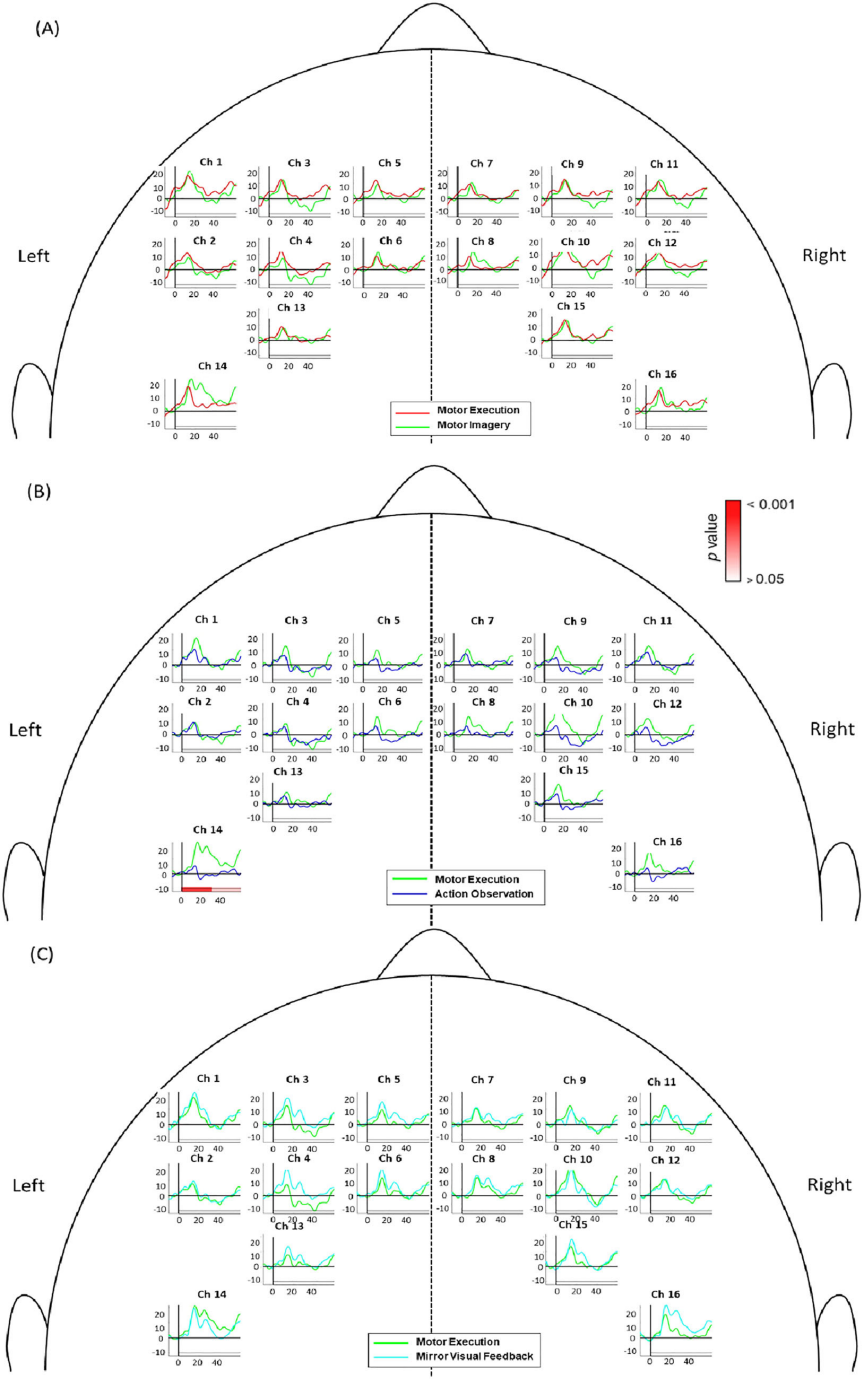

**Figure:**
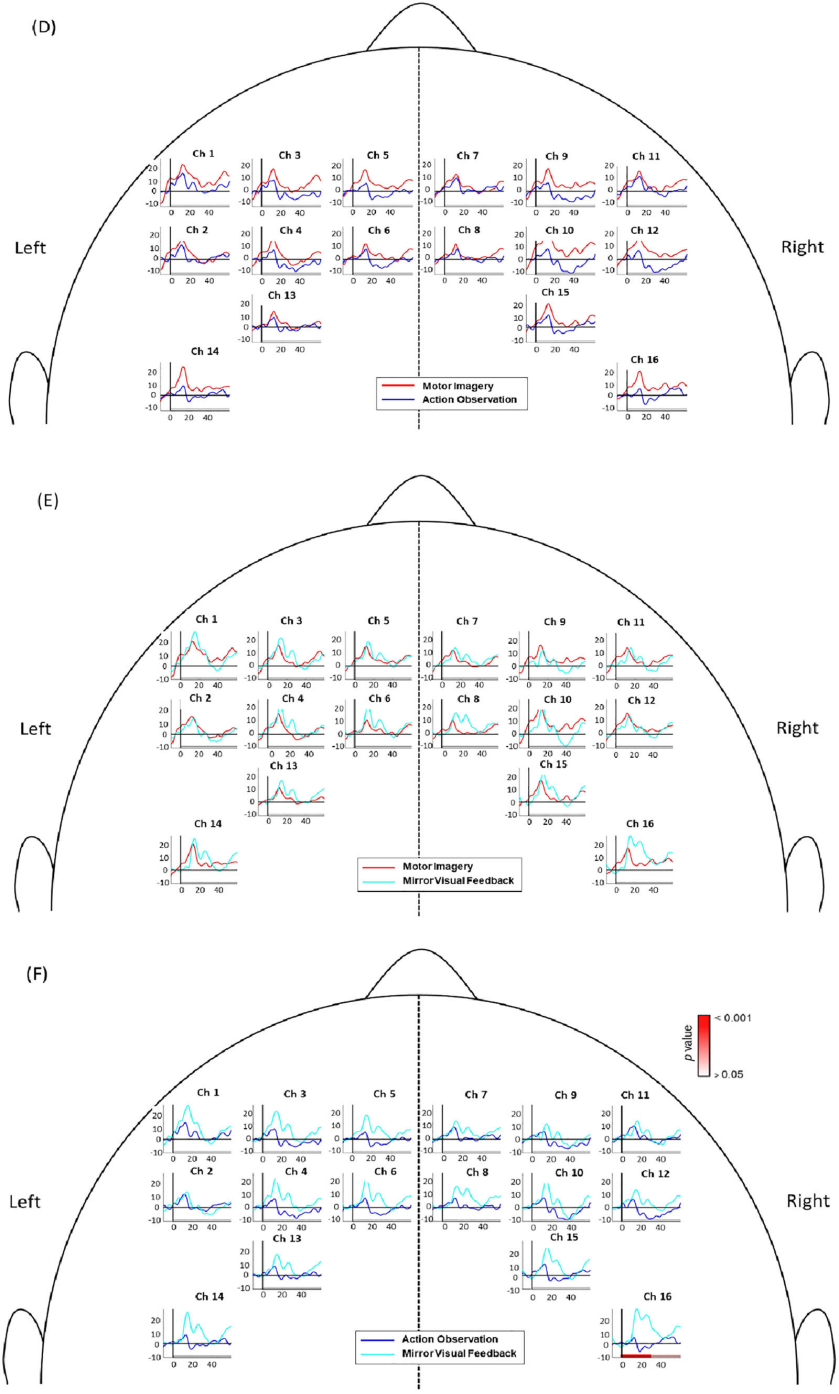

Figure 5B compares the brain activation patterns between ME and AO. Almost all channels during ME exhibited greater activation than during AO. A higher activation level was seen in the right PMC (Ch9, Ch10, Ch11, and Ch12) and left M1 (Ch14) under ME compared to AO. After the statistical analysis, the activation in left M1 (Ch14) showed statistically significant higher activation than AO in the early phase after task onset (*p* = 0.008).

Figure 5D,E compare the brain activation patterns between MI and AO and MI and MVF, respectively. All channels during MI exhibited greater activation compared to AO; however, none of the channels were statistically significant. Multiple channels appeared to exhibit a higher level and a longer duration of brain activation under MVF compared to MI (Figure 5E). Following statistical analyses, however, no significant differences were found in activation patterns between MI and MVF.

Comparing the results of brain activation between AO and MVF, results showed that all the channels exhibited greater activation trends during MVF in the early phase after task onset (Figure 5F). During MVF, the right M1 (Ch16) showed significant difference (*p* < 0.001) in activation as compared to during AO  .

**TABLE 1:** Brain activation during early phase (between 0 and 30 s after task onset) indicated by HbO level in different channels under four motor acquisition tasks.

Brain area	ME	MI	AO	MVF
L′t PMC (Ch1)	**0.11 ± 0.10**	**0.12 ± 0.20**	0.08 ± 0.13	**0.14 ± 0.21**
L′t PMC (Ch2)	0.24 ± 0.12	0.28 ± 0.15	0.25 ± 0.11	0.26 ± 0.12
L′t PMC (Ch3)	0.09 ± 0.14	0.12 ± 0.18	0.07 ± 0.09	**0.15 ± 0.15**
L′t PMC (Ch4)	0.26 ± 0.12	0.30 ± 0.16	0.25 ± 0.11	**0.34 ± 0.16**
L′t SMA (Ch5)	0.12 ± 0.08	**0.15 ± 0.12**	0.09 ± 0.11	**0.17 ± 0.11**
L′t SMA (Ch6)	0.36 ± 0.10	0.36 ± 0.10	0.32 ± 0.10	**0.39 ± 0.12**
R′t SMA (Ch7)	**0.16 ± 0.09**	**0.18 ± 0.11**	0.15 ± 0.11	**0.19 ± 0.10**
R′t SMA (Ch8)	**0.40 ± 0.08**	0.38 ± 0.10	0.37 ± 0.09	**0.42 ± 0.10**
R′t PMC (Ch9)	0.21 ± 0.14	**0.22 ± 0.13**	0.16 ± 0.10	0.19 ± 0.11
R′t PMC (Ch10)	**0.48 ± 0.14**	**0.48 ± 0.17**	0.39 ± 0.12	0.45 ± 0.18
R′t PMC (Ch11)	0.24 ± 0.16	0.25 ± 0.14	0.23 ± 0.15	0.24 ± 0.13
R′t PMC (Ch12)	**0.46 ± 0.10**	**0.49 ± 0.13**	0.41 ± 0.10	**0.47 ± 0.13**
L′t M1 (Ch13)	0.32 ± 0.11	0.32 ± 0.10	0.30 ± 0.09	**0.35 ± 0.13**
L′t M1 (Ch14)	**0.60 ± 0.13**	0.55 ± 0.19	0.49 ± 0.14*	**0.56 ± 0.18**
R′t M1 (Ch15)	**0.50 ± 0.09**	**0.51 ± 0.12**	0.46 ± 0.10	**0.53 ± 0.16**
R′t M1 (Ch16)	**0.57 ± 0.14**	**0.58 ± 0.15**	0.51 ± 0.13**	**0.62 ± 0.15**

*Note*: Values are mean ± SD. Bold text, significant increases in brain activation under each task.

Abbreviations: AO, action observation; HbO, concentration of hemoglobin; L′t, left; M1, primary motor cortex; ME, motor execution; MI, motor imagery; MVF, mirror visual feedback; PMC, premotor cortex; R′t, right; SMA, supplementary area.

* *p* < 0.05 as compared with ME.

** *p* < 0.05 as compared with MVF.

## 4 Discussion

This study investigated brain activity during a forward reaching and grasping task across four different modes of motor acquisition. To our knowledge, this is one of the first studies to use fNIRS to systematically compare activation patterns across these modes, which have been previously studied individually. Moreover, our study uniquely incorporated a functional everyday task and included a systematic comparison of all four modes, including MVF. Understanding the activation patterns is crucial for clinical applications.

During ME of the forward‐reaching and grasping task, the bilateral M1, significant activation was observed in the bilateral M1, particularly the M1 contralateral to the moving limb (Figure 5A). ME involves sensory, proprioceptive, and visual feedback to drive goal‐oriented motor actions through top‐down commands from the M1 via the corticospinal tract (Scott 2016). Our findings reflect the lateralization of motor control during self‐initiated movement, wherein the contralateral motor cortex is primarily activated, with limited activation in ipsilateral regions (An et al. 2013; Colebatch et al. 1991). Prior studies found that ME of a unilateral task induces brain activity contralateral to the moving limb; however, our findings for ME also indicated significant brain activity in several channels on the ipsilateral M1 during the early phase after task onset (Condy et al. 2021). This ipsilateral activation may be attributed to our incorporation of the functional forward reaching and grasping task. Previous research on ME of distal movements, such as mass grasping, finger pinching, or finger tapping, typically demonstrate greater activation confined to the brain areas contralateral to the moving limb. (McCrea, Eng, and Hodgson 2002) In contrast, our forward‐reaching task involves coordinating multiple joints and maintaining postural control. Participants were instructed to sit upright while performing the reaching task, which was accompanied by a distal grasping movement. The combination of maintaining posture, stabilizing the proximal shoulder for reaching, and performing distal hand movements may explain the increased activity in the ipsilateral M1 and other related regions such as the PMC and SMA (Bundy and Leuthardt 2019).

Significant activation was also observed in bilateral brain areas under MI and MVF. MI involves imagining an action without actual movement, emphasizing movement planning, processing, preparation, and inhibition, and engages the SMA and PMC (Hetu et al. 2013; Kasess et al. 2008). Although some studies have reported inconsistent M1 activation during MI, possibly due to inhibitory effects of an active SMA, our results align with previous findings showing smaller M1 activation during MI compared to ME (An et al. 2013; Kasess et al. 2008; Sitaram et al. 2007). During MVF, our results revealed significant activation in all brain regions of interest. Specifically, more regions of the left PMC (Ch1, Ch3, Ch4) showed stronger activation compared to the right PMC (Ch12) (Figure 5D). Participants executed the functional reaching task with their left arm, imagining it was their right arm performing the movement. MVF thus involves aspects of both visual feedback as well as of MI, which potentially explains the high degree of involvement in the PMC and SMA, which are also highly active during MI and AO (Park et al. 2015). The vivid observation facilitated by the mirror, combined with the MI of the mirrored visual illusion, likely contributes to the increased brain activation on the side ipsilateral to the moving limb. Previous studies have shown that MVF activates the PMC on the mirror side, potentially due to the PMC's role in the mirror neuron system (MNS) and its involvement in planning and processing (Qiu et al. 2022; Grezes and Decety 2001; Ding et al. 2018). Thus, MVF can be considered a special type of bilateral movement (Deconinck et al. 2016).

Previous fNIRS studies that examined brain activity during AO found significant bilateral activation (Miguel et al. 2021). Specifically, the SMA, the PMC, the temporal gyri, and other somatosensory regions showed significant increases in activity in adults when observing someone perform an action (Miguel et al. 2021). Brain regions involved in AO have been found to overlap with those involved in MI, including the PMC, pre‐SMA, and parietal regions (Hardwick et al. 2018). Furthermore, during AO, the MNS in the PMC is activated to facilitate observation and subsequent imitation of the action (Caspers et al. 2010). The results of our study, however, indicated that although there was a trend of increasing brain activity during the early phase of the AO task, none of the channels demonstrated significant activation. Our findings do not reflect those from previous studies that indicated significant bilateral activation during AO (Condy et al. 2021). This discrepancy may be due to differences in methodology. For instance, Ge et al. (2018) reported that observing actions from a first‐person perspective caused more extensive activation in key MNS areas compared to a third‐person perspective. Although our study used a first‐person perspective video, the activation levels did not reach statistical significance, possibly due to the reduced screen size and distance from the participant, which may have affected self‐recognition. Furthermore, participants were not instructed to imagine themselves in the first‐person perspective, potentially leading to lower brain activation throughout the task.

Our study compared the brain activation patterns across the four modes of motor acquisition (ME vs. MI, ME vs. AO, ME vs. MVF, MI vs. AO, MI vs. MVF, AO vs. MVF). Although an upward trend in activation could be seen in all the regions of interest in each comparison, only significant differences in brain activation could be found in ME versus AO and AO versus MVF (Figure 5B,F). Notably, the left M1 exhibited significantly greater activation during ME compared to AO (Figure 5B). AO induced less brain activation compared to MI and MVF, consistent with recent findings that brain lateralization is unaffected when observing another person's actions (Khaksari et al. 2021). Compared to the other three modes, AO showed a relatively quick decrease in brain activity even in the early phase, possibly due to lower attentional demands compared to other modes. The task's low difficulty and repetitive nature might have limited its ability to induce significant brain activity. Both MI and AO involved no actual movement, which might explain the reduced M1 activation compared to ME and MVF.

Significant brain activation was observed in the right M1 when comparing AO and MVF modes. MI and MVF exhibited greater similarities in brain activation patterns compared to motor ME. The duration of brain activation in the bilateral M1 during MVF lasted longer than in MI, resembling the activation patterns of ME. This is noteworthy given the different nature of ME (actual execution by the right arm) and MVF (execution by the contralateral limb). The ability of MVF to induce similar and more sustained activation compared to MI holds promise for future clinical applications targeting neuroplasticity.

Although participants completed the 60‐s functional reaching task in each block, brain activation in most channels was prominent mainly within the initial 30 s after task onset. After the 30‐s mark, brain activity levels decreased, returning to baseline values. The low difficulty and repetitive nature of the task could explain these findings. For healthy young subjects aged 20–30, the repetitive forward‐reaching movement of the dominant hand is not challenging. The significant activation after task onset suggests initial task adjustment, with subsequent repetitions becoming automatic and requiring fewer attentional resources.

### 4.1 Significance of the Findings and Clinical Applications

This study provides insights into how different motor acquisition modes, ME, MI, AO, and MVF, affect brain activation patterns during a functional reaching and grasping task. Our findings reveal that MI and MVF induced activation patterns similar to ME, suggesting their potential as effective alternatives in clinical settings where actual movement may not be feasible.

Specifically, MI recruited more brain regions associated with motor planning and processing compared to ME, while MVF exhibited significant activation in a broader range of brain regions and showed a longer activation duration in the M1 region of the mirrored limb. The sustained activation seen in MVF, particularly in the ipsilateral motor pathways, holds promise for neuroplasticity interventions in patients with neurological impairments such as stroke (Deconinck et al. 2016; Rjosk et al. 2017).

Integrating MI and MVF into rehabilitation protocols can potentially enhance motor recovery and functional outcomes, making them valuable options when ME is not possible to perform. For patients with hemiparesis or difficulty with both upper extremities, MVF and MI can be considered as treatment interventions to support brain plasticity and recovery. This study underscores the importance of selecting appropriate motor acquisition modes based on individual patient needs, paving the way for more personalized and effective rehabilitation strategies.

### 4.2 Limitations

The present study has several limitations. First, the participants were young, healthy adults, and future research should include diverse populations, such as the elderly, individuals with stroke, and those with neurological diseases. Second, the study focused on right‐handed individuals, and incorporating left‐handed participants and using a more rigorous measure of handedness, such as the Edinburgh Handedness Inventory, would provide a fuller understanding. Third, due to instrumental constraints, the study only observed activation patterns in specific brain areas. Future research may be needed to explore activation in a broader range of brain regions during these motor acquisition modes, especially in patient populations. Fourth, the study did not include detailed spatial coordinate analysis, such as MNI coordinates, which could limit spatial precision and comparability. Future research should incorporate standardized spatial coordinates for better spatial accuracy. Fifth, our study is the lack of direct measurement of MI accuracy. Although we assumed that healthy university students could accurately understand and execute the imagery task based on the provided instructions, this assumption introduces a potential source of error. In addition, we did not assess the vividness of MI, which might be an explanatory variable influencing the results. Finally, the relatively low temporal resolution of fNIRS may have limited our ability to capture rapid neural dynamics, which could affect the interpretation of the timing and sequence of brain activations. Combining fNIRS with EEG in future research could enhance temporal resolution and provide more detailed insights into neural activity (Su et al. 2023).

## 5 Conclusions

This study utilized fNIRS to compare brain activation patterns during a functional task across ME, MI, AO, and MVF. The results indicated that different motor acquisition modes exert distinct brain activation patterns. Specifically, activation levels under MI and MVF were comparable to ME. MI and MVF induced greater activity in the PMC and SMA, with MVF showing significant activity in all brain areas, especially in the bilateral M1. These findings enhance our understanding of the neural basis of different motor acquisition modes, which is essential for training and clinical rehabilitation.

## Author Contributions

**Meng‐Hsuan Tsou**: writing–original draft, visualization, formal analysis, data curation. **Pei‐Yun Chen**: conceptualization, methodology, data curation, formal analysis, project administration, visualization. **Yi‐Ting Hung**: visualization, validation, investigation, data curation. **Yong‐Wei Lim**: formal analysis, visualization, investigation. **Shiuan‐Ling Huang**: formal analysis, visualization, investigation. **Yan‐Ci Liu**: conceptualization, methodology, supervision, resources, project administration, visualization, writing–review and editing, validation, formal analysis, software.

## Ethics Statement

This study protocol was approved by the National Taiwan University Hospital Institutional Review Board (NTUH IRB).

## Consent

The participant in Figure 1 approved of the use of the images in this manuscript.

## Conflicts of Interest

The authors declare no conflicts of interest.

### Peer Review

The peer review history for this article is available at https://publons.com/publon/10.1002/brb3.70238.

## Data Availability

The datasets used and/or analyzed during the current study are available from the corresponding author on reasonable request.
